# Supplementary material for: Predicting visual function by interpreting a neuronal wiring diagram
Source: Nature. 2024 Oct 2;634(8032):113–23. doi: 10.1038/s41586-024-07953-5 (PMC11446822; doi:10.1038/s41586-024-07953-5)
Supplement: Supplementary file 5 — CRF and ERF predictions for individual Dm3 cells. The CRF is predicted for each target cell by mapping the top five monosynaptic pathways from hexel source types to the target Dm3 type. The ERF is predicted using disynaptic pathways that pass through the top ten intermediary types (ranking from Extended Data Fig. 7). As a set of disynaptic pathways passes from multiple hexel source types through each intermediary to the target, for brevity only one representative starting from a single hexel type is shown, the pathway in the set with maximal strength (red line, Extended Data Fig. 7). The centre of the Tm1–Dm3 CRF (Methods) is indicated by the cyan star, to aid comparison of locations across maps. Next to each map are the maximum and sum over all hexels, in units of 0.01%. In the last panel, all ellipse approximations are shown together for comparison, scaled up by ×3 relative to maps. The dashed ellipse approximates the Tm1–Dm3 CRF, and scale bars indicate one lattice constant and p and q axes. Each file name contains the ID of the target cell in v783. [file 41586_2024_7953_MOESM5_ESM.zip › DataS3/Dm3v/720575940606729150.pdf]

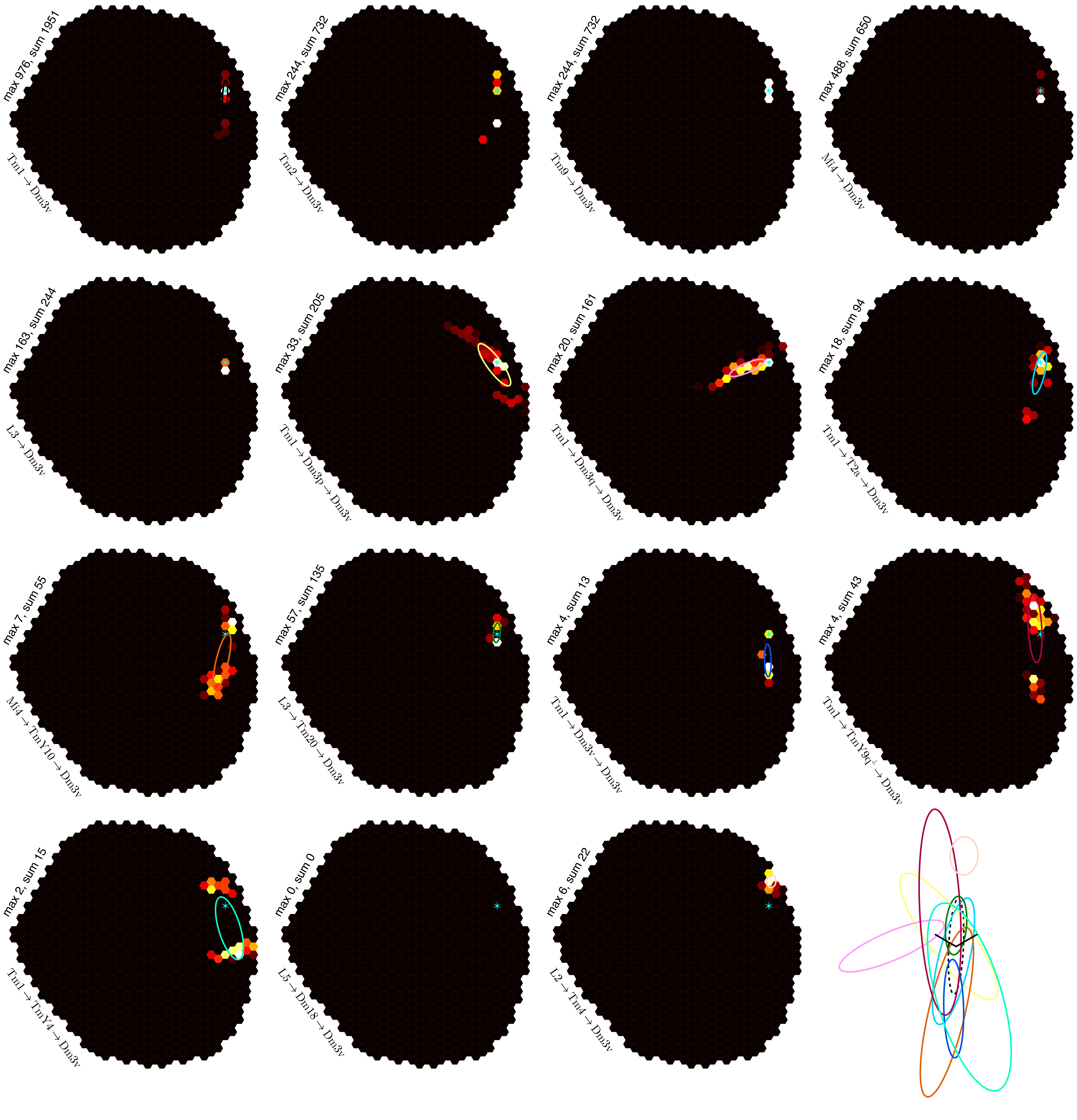
max 976, sum 1951
Tm1 → Dm3v
max 244, sum 732
Tm2 → Dm3v
max 244, sum 732
Tm9 → Dm3v
max 488, sum 650
Mi4 → Dm3v
max 163, sum 244
L3 → Dm3v
max 33, sum 205
Tm1 → Dm3p → Dm3v
max 20, sum 161
Tm1 → Dm3q → Dm3v
max 18, sum 94
Tm1 → T2a → Dm3v
max 7, sum 55
Mi4 → TmY10 → Dm3v
max 57, sum 135
L3 → Tm20 → Dm3v
max 4, sum 13
Tm1 → Dm3v → Dm3v
max 4, sum 43
Tm1 → TmY9q⊥ → Dm3v
max 2, sum 15
Tm1 → TmY4 → Dm3v
max 0, sum 0
L5 → Dm18 → Dm3v
max 6, sum 22
L2 → Tm4 → Dm3v
